# Bis[4-(dimethyl­amino)­pyridinium] bis­[4-(dimethyl­amino)­pyridine-κ*N* ^1^]tetra­kis­(thio­cyanato-κ*N*)manganate(II)

**DOI:** 10.1107/S1600536812047678

**Published:** 2012-11-24

**Authors:** Susanne Wöhlert, Inke Jess, Christian Näther

**Affiliations:** a Institut für Anorganische Chemie, Christian-Albrechts-Universität Kiel, Max-Eyth-Strasse 2, 24118 Kiel, Germany

## Abstract

In the crystal structure of the title compound, (C_7_H_11_N_2_)_2_[Mn(NCS)_4_(C_7_H_10_N_2_)_2_], the manganese(II) cations are coordinated by four *N*-bonded thio­cyanate anions and two *N*-bonded 4-(dimethyl­amino)­pyridine ligands into discrete complex dianions. For charge balance, two 4-(dimethylamino)pyridine counter cations are present, which do not coordinate to the metal cation. The asymmetric unit consists of one manganese(II) cation, four thio­cyanate anions and two 4-(dimethyl­amino)­pyridine ligands, as well as two protonated 4-(dimethyl­amino)­pyridinium cations. The discrete complex anions are connected to the non-coordinating pyridinium cations by weak N—H⋯S hydrogen-bonding inter­actions.

## Related literature

For general background, see: Boeckmann & Näther (2011 ▶, 2012 ▶).
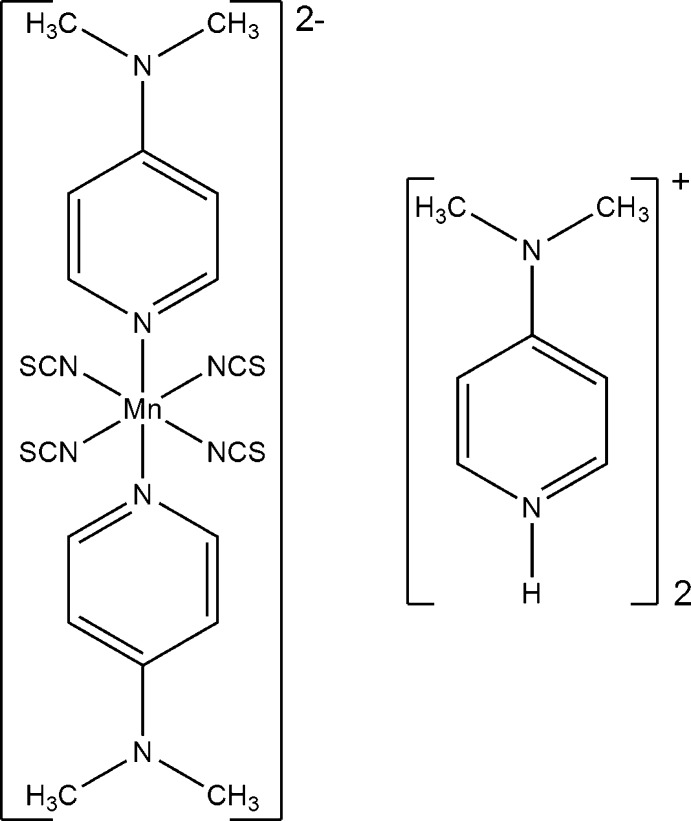


## Experimental

### 

#### Crystal data

(C_7_H_11_N_2_)_2_[Mn(NCS)_4_(C_7_H_10_N_2_)_2_]
*M* *_r_* = 777.96
Triclinic, 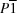

*a* = 11.7307 (8) Å
*b* = 11.9010 (9) Å
*c* = 15.4224 (12) Å
α = 102.520 (9)°
β = 96.794 (9)°
γ = 107.400 (8)°
*V* = 1966.6 (3) Å^3^
*Z* = 2
Mo *K*α radiation
μ = 0.59 mm^−1^
*T* = 180 K
0.18 × 0.11 × 0.05 mm

#### Data collection

Stoe IPDS-1 diffractometer
Absorption correction: numerical (*X-SHAPE* and *X-RED32*; Stoe & Cie, 2008 ▶) *T*_min_ = 0.873, *T*_max_ = 0.968
24452 measured reflections
9338 independent reflections
6752 reflections with *I* > 2σ(*I*)
*R*_int_ = 0.041

#### Refinement

*R*[*F* ^2^ > 2σ(*F* ^2^)] = 0.044
*wR*(*F* ^2^) = 0.121
*S* = 1.02
9338 reflections
450 parameters
H-atom parameters constrained
Δρ_max_ = 0.80 e Å^−3^
Δρ_min_ = −0.72 e Å^−3^

### 

Data collection: *X-AREA* (Stoe & Cie, 2008 ▶); cell refinement: *X-AREA*; data reduction: *X-AREA*; program(s) used to solve structure: *SHELXS97* (Sheldrick, 2008 ▶); program(s) used to refine structure: *SHELXL97* (Sheldrick, 2008 ▶); molecular graphics: *XP* in *SHELXTL* (Sheldrick, 2008 ▶) and *DIAMOND* (Brandenburg, 2012 ▶); software used to prepare material for publication: *XCIF* in *SHELXTL*.

## Supplementary Material

Crystal structure: contains datablock(s) I, global. DOI: 10.1107/S1600536812047678/vm2182sup1.cif

Structure factors: contains datablock(s) I. DOI: 10.1107/S1600536812047678/vm2182Isup2.hkl

Additional supplementary materials:  crystallographic information; 3D view; checkCIF report

## Figures and Tables

**Table 1:** Selected bond lengths (Å)

Mn1—N1	2.1928 (17)
Mn1—N2	2.2014 (19)
Mn1—N3	2.2468 (19)
Mn1—N4	2.2535 (18)
Mn1—N20	2.2561 (16)
Mn1—N10	2.2659 (16)

**Table 2:** Hydrogen-bond geometry (Å, °)

*D*—H⋯*A*	*D*—H	H⋯*A*	*D*⋯*A*	*D*—H⋯*A*
N30—H30⋯S4	0.88	2.36	3.217 (2)	165
N40—H40*A*⋯S3^i^	0.88	2.35	3.213 (3)	166

Symmetry code: (i) 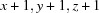.

**Fig. 1.**
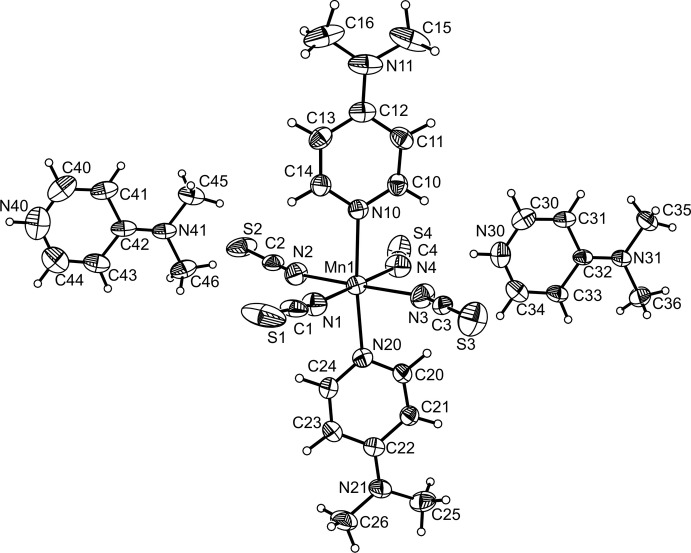
Crystal structure of the title compound with labeling and displacement ellipsoids drawn at the 50% probability level.

**Fig. 2.**
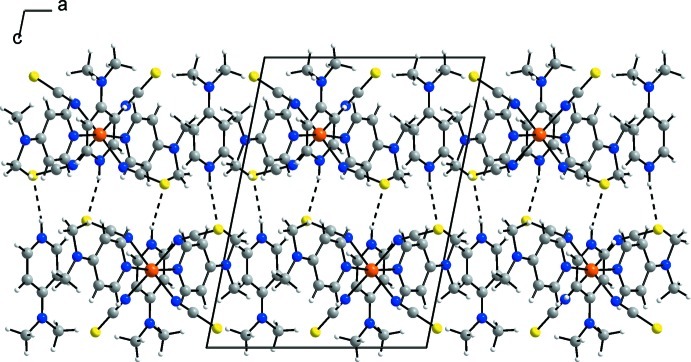
Crystal structure of the title compound with view along the crystallographic b-axis. Intermolecular hydrogen bonding is shown as dashed lines.

## supplementary crystallographic information

### Comment

The structure determination was performed during a project on the synthesis, thermal and magnetic properties of coordination compounds based on transition metal thiocyanates and neutral N-donor co-ligands (Boeckmann & Näther, 2011 & 2012). In order to investigate the influence of the co-ligand, N,N'-dimethylaminopyridine was reacted with manganese(II) thiocyanate which resulted in the formation of crystals of the title compound that were identified by single-crystal X-ray diffraction.

In the crystal structure of the title compound each manganese(II) cation is coordinated by four N-bonded thiocyanato anions and two N-bonded dimethylaminopyridine ligands (Fig. 1). The MnN_6_ octahedra are slightly distorted with distances in the range of 2.1928 (19) Å to 2.2659 (16) Å (Table 1). The angles arround the manganese(II) cations are ranging from 87.73 (6)° to 92.86 (7) ° and 173.95 (6) ° to 178.78 (7) ° (Table 1). There are additional protonated dimethylaminopyridine ligands that does not coordinate to the metal cations, but which are linked to the complex cations by weak intermolecular N–H···S hydrogen bonding, which are ranging from 3.213 (3) Å (N40-H40A···S3[1 + *x*, 1 + *y*, 1 + *z*]) to 3.217 (2) Å (N30-H30···S4, see Fig. 2).

### Experimental

MnSO_4_xH_2_O, Ba(NCS)_2_x3H_2_O and N,N'-dimethylaminopyridine were obtained from Sigma Aldrich. 0.15 mmol (26 mg) Mn(NCS)_2_ and 0.3 mmol (58.3 mg) dimethylaminopyridine were reacted with 1 mL ethanol in a snap cap vial. After three days yellow colored block-shaped single crystals of the title compound were obtained.

### Refinement

All C-H and N-H H atoms were located in difference map but were positioned with idealized geometry (methyl H atoms allowed to rotate but not to tip) and were refined isotropic with *U*_iso_(H) = 1.2 *U*_eq_(C, N) (1.5 for the methyl H atoms) using a riding model with C_aromatic_ = 0.95 Å, CmethylH = 0.98 Å and N—H = 0.88 Å.

### Crystal data

**Table:**

(C_7_H_11_N_2_)_2_[Mn(NCS)_4_(C_7_H_10_N_2_)_2_]	*Z* = 2
*M**_r_* = 777.96	*F*(000) = 814
Triclinic, *P*1	*D*_x_ = 1.314 Mg m^−^^3^
Hall symbol: -P 1	Mo *K*α radiation, λ = 0.71073 Å
*a* = 11.7307 (8) Å	Cell parameters from 24452 reflections
*b* = 11.9010 (9) Å	θ = 2.6–28.0°
*c* = 15.4224 (12) Å	µ = 0.59 mm^−^^1^
α = 102.520 (9)°	*T* = 180 K
β = 96.794 (9)°	Block, yellow
γ = 107.400 (8)°	0.18 × 0.11 × 0.05 mm
*V* = 1966.6 (3) Å^3^	

### Data collection

**Table:**

Stoe IPDS-1 diffractometer	9338 independent reflections
Radiation source: fine-focus sealed tube	6752 reflections with *I* > 2σ(*I*)
Graphite monochromator	*R*_int_ = 0.041
phi scan	θ_max_ = 28.0°, θ_min_ = 2.6°
Absorption correction: numerical (*X-SHAPE* and *X-RED32*; Stoe & Cie, 2008)	*h* = −15→15
*T*_min_ = 0.873, *T*_max_ = 0.968	*k* = −15→15
24452 measured reflections	*l* = −20→20

### Refinement

**Table:**

Refinement on *F*^2^	Primary atom site location: structure-invariant direct methods
Least-squares matrix: full	Secondary atom site location: difference Fourier map
*R*[*F*^2^ > 2σ(*F*^2^)] = 0.044	Hydrogen site location: inferred from neighbouring sites
*wR*(*F*^2^) = 0.121	H-atom parameters constrained
*S* = 1.02	*w* = 1/[σ^2^(*F*_o_^2^) + (0.0727*P*)^2^ + 0.0822*P*] where *P* = (*F*_o_^2^ + 2*F*_c_^2^)/3
9338 reflections	(Δ/σ)_max_ = 0.001
450 parameters	Δρ_max_ = 0.80 e Å^−^^3^
0 restraints	Δρ_min_ = −0.72 e Å^−^^3^

### Special details

**Table:**

Geometry. All esds (except the esd in the dihedral angle between two l.s. planes) are estimated using the full covariance matrix. The cell esds are taken into account individually in the estimation of esds in distances, angles and torsion angles; correlations between esds in cell parameters are only used when they are defined by crystal symmetry. An approximate (isotropic) treatment of cell esds is used for estimating esds involving l.s. planes.
Refinement. Refinement of F^2^ against ALL reflections. The weighted R-factor wR and goodness of fit S are based on F^2^, conventional R-factors R are based on F, with F set to zero for negative F^2^. The threshold expression of F^2^ > 2sigma(F^2^) is used only for calculating R-factors(gt) etc. and is not relevant to the choice of reflections for refinement. R-factors based on F^2^ are statistically about twice as large as those based on F, and R- factors based on ALL data will be even larger.

### Fractional atomic coordinates and isotropic or equivalent isotropic displacement parameters (Å^2^)

**Table:**

	*x*	*y*	*z*	*U*_iso_*/*U*_eq_	
Mn1	0.68016 (2)	0.43208 (2)	0.733534 (19)	0.03223 (9)	
N1	0.58727 (17)	0.35462 (17)	0.83317 (13)	0.0465 (4)	
C1	0.5433 (2)	0.29723 (19)	0.87948 (16)	0.0449 (5)	
S1	0.48122 (9)	0.21875 (8)	0.94635 (7)	0.0889 (3)	
N2	0.81931 (17)	0.57958 (16)	0.84005 (13)	0.0479 (4)	
C2	0.90686 (19)	0.65777 (17)	0.87884 (14)	0.0387 (4)	
S2	1.03117 (6)	0.76864 (5)	0.93376 (6)	0.0679 (2)	
N3	0.54216 (18)	0.28333 (16)	0.62122 (14)	0.0516 (5)	
C3	0.45842 (18)	0.19649 (17)	0.59757 (13)	0.0363 (4)	
S3	0.33917 (6)	0.07240 (6)	0.56332 (5)	0.0677 (2)	
N4	0.77439 (18)	0.50766 (18)	0.62913 (14)	0.0507 (5)	
C4	0.84494 (19)	0.58781 (17)	0.61404 (13)	0.0364 (4)	
S4	0.94638 (7)	0.70095 (6)	0.59289 (5)	0.0673 (2)	
N10	0.79695 (14)	0.31067 (13)	0.72983 (11)	0.0344 (3)	
C10	0.81667 (19)	0.25345 (17)	0.65092 (14)	0.0392 (4)	
H10	0.7793	0.2649	0.5969	0.047*	
C11	0.8868 (2)	0.17989 (17)	0.64293 (15)	0.0429 (5)	
H11	0.8969	0.1422	0.5847	0.052*	
C12	0.94409 (18)	0.15981 (16)	0.72082 (16)	0.0416 (5)	
C13	0.92381 (19)	0.21962 (18)	0.80340 (15)	0.0418 (5)	
H13	0.9598	0.2103	0.8587	0.050*	
C14	0.85175 (18)	0.29156 (17)	0.80394 (14)	0.0379 (4)	
H14	0.8398	0.3307	0.8610	0.045*	
N11	1.01300 (19)	0.08574 (18)	0.71490 (18)	0.0611 (6)	
C15	1.0336 (3)	0.0294 (2)	0.6272 (3)	0.0813 (10)	
H15A	0.9558	−0.0266	0.5891	0.122*	
H15B	1.0894	−0.0161	0.6356	0.122*	
H15C	1.0694	0.0930	0.5977	0.122*	
C16	1.0718 (3)	0.0642 (3)	0.7951 (3)	0.0855 (11)	
H16A	1.1449	0.1348	0.8250	0.128*	
H16B	1.0949	−0.0089	0.7775	0.128*	
H16C	1.0153	0.0522	0.8369	0.128*	
N20	0.56112 (15)	0.54793 (14)	0.72115 (11)	0.0345 (3)	
C20	0.51483 (18)	0.55514 (17)	0.63922 (13)	0.0369 (4)	
H20	0.5362	0.5118	0.5879	0.044*	
C21	0.43956 (19)	0.61995 (17)	0.62432 (13)	0.0365 (4)	
H21	0.4107	0.6209	0.5644	0.044*	
C22	0.40472 (17)	0.68564 (16)	0.69838 (13)	0.0346 (4)	
C23	0.45337 (18)	0.67870 (16)	0.78447 (13)	0.0359 (4)	
H23	0.4343	0.7210	0.8374	0.043*	
C24	0.52809 (17)	0.61082 (16)	0.79141 (13)	0.0348 (4)	
H24	0.5590	0.6079	0.8504	0.042*	
N21	0.32914 (17)	0.74991 (16)	0.68615 (13)	0.0444 (4)	
C25	0.2838 (2)	0.7546 (2)	0.59538 (18)	0.0538 (6)	
H25A	0.3527	0.7902	0.5682	0.081*	
H25B	0.2312	0.8051	0.5989	0.081*	
H25C	0.2371	0.6718	0.5580	0.081*	
C26	0.2942 (2)	0.8181 (2)	0.76234 (19)	0.0584 (6)	
H26A	0.2535	0.7623	0.7962	0.088*	
H26B	0.2384	0.8571	0.7401	0.088*	
H26C	0.3670	0.8808	0.8022	0.088*	
N30	0.84902 (18)	0.59839 (17)	0.37737 (13)	0.0502 (5)	
H30	0.8663	0.6130	0.4367	0.060*	
C30	0.9012 (2)	0.5296 (2)	0.32567 (17)	0.0483 (5)	
H30A	0.9558	0.4972	0.3539	0.058*	
C31	0.87713 (18)	0.50555 (17)	0.23413 (15)	0.0392 (4)	
H31	0.9138	0.4557	0.1987	0.047*	
C32	0.79728 (17)	0.55486 (15)	0.19124 (13)	0.0333 (4)	
C33	0.74295 (19)	0.62532 (17)	0.24890 (15)	0.0405 (4)	
H33	0.6869	0.6585	0.2236	0.049*	
C34	0.7712 (2)	0.64492 (19)	0.33973 (16)	0.0474 (5)	
H34	0.7351	0.6927	0.3776	0.057*	
N31	0.77449 (17)	0.53660 (16)	0.10194 (12)	0.0436 (4)	
C35	0.8320 (3)	0.4662 (2)	0.04314 (17)	0.0598 (6)	
H35A	0.8009	0.3803	0.0438	0.090*	
H35B	0.9205	0.4984	0.0650	0.090*	
H35C	0.8133	0.4727	−0.0188	0.090*	
C36	0.6889 (3)	0.5836 (2)	0.05802 (17)	0.0583 (6)	
H36A	0.7067	0.6697	0.0892	0.087*	
H36B	0.6056	0.5366	0.0606	0.087*	
H36C	0.6966	0.5761	−0.0054	0.087*	
N40	1.3442 (2)	1.0729 (2)	1.35554 (18)	0.0741 (7)	
H40A	1.3569	1.0786	1.4140	0.089*	
C40	1.3919 (2)	1.0029 (2)	1.3001 (2)	0.0652 (8)	
H40	1.4394	0.9611	1.3249	0.078*	
C41	1.37352 (19)	0.99125 (19)	1.21042 (19)	0.0495 (6)	
H41	1.4083	0.9419	1.1726	0.059*	
C42	1.30213 (17)	1.05255 (16)	1.17213 (17)	0.0414 (5)	
C43	1.2558 (2)	1.12694 (19)	1.23391 (19)	0.0509 (6)	
H43	1.2093	1.1717	1.2120	0.061*	
C44	1.2772 (3)	1.1342 (2)	1.3222 (2)	0.0644 (7)	
H44	1.2448	1.1834	1.3624	0.077*	
N41	1.28055 (16)	1.04136 (15)	1.08378 (14)	0.0445 (4)	
C45	1.3279 (2)	0.9643 (2)	1.02188 (19)	0.0555 (6)	
H45A	1.4171	0.9935	1.0382	0.083*	
H45B	1.3027	0.9675	0.9598	0.083*	
H45C	1.2959	0.8799	1.0259	0.083*	
C46	1.2060 (2)	1.1027 (2)	1.0441 (2)	0.0581 (6)	
H46A	1.1233	1.0732	1.0556	0.087*	
H46B	1.2030	1.0853	0.9786	0.087*	
H46C	1.2416	1.1911	1.0714	0.087*	

### Atomic displacement parameters (Å^2^)

**Table:**

	*U*^11^	*U*^22^	*U*^33^	*U*^12^	*U*^13^	*U*^23^
Mn1	0.02959 (15)	0.02651 (13)	0.03901 (16)	0.00659 (10)	0.00554 (11)	0.01014 (11)
N1	0.0466 (10)	0.0481 (10)	0.0521 (11)	0.0159 (8)	0.0183 (9)	0.0232 (8)
C1	0.0484 (12)	0.0459 (11)	0.0602 (13)	0.0322 (10)	0.0241 (11)	0.0243 (10)
S1	0.1152 (7)	0.0995 (6)	0.1337 (7)	0.0841 (6)	0.0926 (6)	0.0925 (6)
N2	0.0442 (10)	0.0363 (9)	0.0529 (11)	0.0093 (8)	−0.0018 (9)	0.0028 (8)
C2	0.0388 (11)	0.0325 (9)	0.0478 (11)	0.0203 (8)	0.0054 (9)	0.0069 (8)
S2	0.0375 (3)	0.0424 (3)	0.1037 (6)	0.0146 (2)	−0.0138 (3)	−0.0082 (3)
N3	0.0442 (10)	0.0387 (9)	0.0582 (12)	0.0076 (8)	−0.0057 (9)	0.0025 (8)
C3	0.0367 (10)	0.0362 (9)	0.0368 (10)	0.0177 (8)	0.0022 (8)	0.0065 (8)
S3	0.0525 (4)	0.0541 (3)	0.0742 (4)	−0.0096 (3)	0.0026 (3)	0.0156 (3)
N4	0.0523 (11)	0.0505 (10)	0.0601 (12)	0.0184 (9)	0.0235 (10)	0.0282 (9)
C4	0.0417 (11)	0.0404 (10)	0.0347 (10)	0.0215 (8)	0.0103 (8)	0.0131 (8)
S4	0.0672 (4)	0.0586 (4)	0.0569 (4)	−0.0107 (3)	0.0068 (3)	0.0244 (3)
N10	0.0323 (8)	0.0317 (7)	0.0374 (8)	0.0098 (6)	0.0050 (7)	0.0079 (6)
C10	0.0426 (11)	0.0352 (9)	0.0369 (10)	0.0114 (8)	0.0050 (8)	0.0077 (8)
C11	0.0421 (11)	0.0331 (9)	0.0491 (12)	0.0098 (8)	0.0129 (9)	0.0036 (8)
C12	0.0291 (10)	0.0269 (8)	0.0661 (14)	0.0057 (7)	0.0088 (9)	0.0121 (9)
C13	0.0361 (11)	0.0368 (9)	0.0487 (12)	0.0086 (8)	0.0000 (9)	0.0135 (9)
C14	0.0369 (10)	0.0353 (9)	0.0369 (10)	0.0088 (8)	0.0042 (8)	0.0069 (8)
N11	0.0457 (11)	0.0421 (10)	0.1033 (18)	0.0220 (9)	0.0201 (12)	0.0215 (11)
C15	0.0727 (19)	0.0453 (13)	0.139 (3)	0.0292 (13)	0.054 (2)	0.0190 (16)
C16	0.0543 (17)	0.0700 (18)	0.142 (3)	0.0330 (14)	0.0018 (18)	0.041 (2)
N20	0.0338 (8)	0.0320 (7)	0.0382 (8)	0.0111 (6)	0.0060 (7)	0.0107 (6)
C20	0.0408 (11)	0.0368 (9)	0.0343 (9)	0.0159 (8)	0.0083 (8)	0.0070 (7)
C21	0.0402 (11)	0.0379 (9)	0.0340 (9)	0.0160 (8)	0.0068 (8)	0.0109 (8)
C22	0.0323 (10)	0.0278 (8)	0.0435 (10)	0.0082 (7)	0.0100 (8)	0.0103 (7)
C23	0.0382 (10)	0.0307 (8)	0.0371 (10)	0.0084 (7)	0.0127 (8)	0.0071 (7)
C24	0.0349 (10)	0.0330 (9)	0.0333 (9)	0.0064 (7)	0.0053 (8)	0.0103 (7)
N21	0.0481 (10)	0.0421 (9)	0.0509 (10)	0.0260 (8)	0.0120 (8)	0.0115 (8)
C25	0.0541 (14)	0.0552 (13)	0.0665 (15)	0.0310 (11)	0.0122 (12)	0.0274 (12)
C26	0.0582 (15)	0.0530 (13)	0.0718 (17)	0.0335 (12)	0.0189 (13)	0.0078 (12)
N30	0.0524 (11)	0.0491 (10)	0.0413 (10)	0.0029 (9)	0.0119 (9)	0.0147 (8)
C30	0.0394 (11)	0.0479 (11)	0.0606 (14)	0.0107 (9)	0.0073 (10)	0.0273 (11)
C31	0.0345 (10)	0.0361 (9)	0.0534 (12)	0.0169 (8)	0.0105 (9)	0.0169 (8)
C32	0.0302 (9)	0.0258 (8)	0.0453 (11)	0.0112 (7)	0.0079 (8)	0.0096 (7)
C33	0.0387 (11)	0.0315 (9)	0.0534 (12)	0.0157 (8)	0.0130 (9)	0.0080 (8)
C34	0.0473 (12)	0.0365 (10)	0.0551 (13)	0.0085 (9)	0.0217 (10)	0.0071 (9)
N31	0.0505 (11)	0.0422 (9)	0.0446 (10)	0.0286 (8)	0.0063 (8)	0.0079 (7)
C35	0.0777 (18)	0.0674 (15)	0.0462 (13)	0.0473 (14)	0.0131 (12)	0.0064 (11)
C36	0.0701 (17)	0.0613 (14)	0.0520 (13)	0.0410 (13)	−0.0021 (12)	0.0130 (11)
N40	0.0617 (15)	0.0691 (15)	0.0688 (15)	−0.0134 (12)	0.0079 (12)	0.0243 (13)
C40	0.0426 (13)	0.0569 (15)	0.090 (2)	0.0009 (11)	−0.0024 (13)	0.0387 (15)
C41	0.0324 (11)	0.0368 (10)	0.0816 (17)	0.0105 (8)	0.0084 (11)	0.0233 (11)
C42	0.0263 (9)	0.0272 (8)	0.0725 (15)	0.0081 (7)	0.0120 (9)	0.0171 (9)
C43	0.0392 (12)	0.0380 (10)	0.0768 (17)	0.0117 (9)	0.0205 (11)	0.0146 (10)
C44	0.0524 (15)	0.0523 (14)	0.0772 (19)	0.0009 (11)	0.0238 (14)	0.0119 (13)
N41	0.0376 (9)	0.0363 (8)	0.0659 (12)	0.0194 (7)	0.0112 (9)	0.0161 (8)
C45	0.0474 (13)	0.0452 (12)	0.0766 (17)	0.0202 (10)	0.0191 (12)	0.0115 (11)
C46	0.0525 (14)	0.0541 (13)	0.0782 (17)	0.0295 (11)	0.0067 (13)	0.0257 (12)

### Geometric parameters (Å, º)

**Table:**

Mn1—N1	2.1928 (17)	C25—H25A	0.9800
Mn1—N2	2.2014 (19)	C25—H25B	0.9800
Mn1—N3	2.2468 (19)	C25—H25C	0.9800
Mn1—N4	2.2535 (18)	C26—H26A	0.9800
Mn1—N20	2.2561 (16)	C26—H26B	0.9800
Mn1—N10	2.2659 (16)	C26—H26C	0.9800
N1—C1	1.153 (3)	N30—C34	1.339 (3)
C1—S1	1.629 (2)	N30—C30	1.346 (3)
N2—C2	1.149 (3)	N30—H30	0.8800
C2—S2	1.630 (2)	C30—C31	1.355 (3)
N3—C3	1.145 (3)	C30—H30A	0.9500
C3—S3	1.633 (2)	C31—C32	1.418 (3)
N4—C4	1.148 (3)	C31—H31	0.9500
C4—S4	1.631 (2)	C32—N31	1.328 (3)
N10—C14	1.341 (2)	C32—C33	1.425 (3)
N10—C10	1.342 (3)	C33—C34	1.352 (3)
C10—C11	1.366 (3)	C33—H33	0.9500
C10—H10	0.9500	C34—H34	0.9500
C11—C12	1.408 (3)	N31—C36	1.456 (3)
C11—H11	0.9500	N31—C35	1.461 (3)
C12—N11	1.359 (3)	C35—H35A	0.9800
C12—C13	1.402 (3)	C35—H35B	0.9800
C13—C14	1.371 (3)	C35—H35C	0.9800
C13—H13	0.9500	C36—H36A	0.9800
C14—H14	0.9500	C36—H36B	0.9800
N11—C16	1.454 (4)	C36—H36C	0.9800
N11—C15	1.456 (4)	N40—C40	1.349 (4)
C15—H15A	0.9800	N40—C44	1.350 (4)
C15—H15B	0.9800	N40—H40A	0.8800
C15—H15C	0.9800	C40—C41	1.345 (4)
C16—H16A	0.9800	C40—H40	0.9500
C16—H16B	0.9800	C41—C42	1.420 (3)
C16—H16C	0.9800	C41—H41	0.9500
N20—C24	1.344 (3)	C42—N41	1.326 (3)
N20—C20	1.346 (2)	C42—C43	1.425 (3)
C20—C21	1.365 (3)	C43—C44	1.335 (4)
C20—H20	0.9500	C43—H43	0.9500
C21—C22	1.413 (3)	C44—H44	0.9500
C21—H21	0.9500	N41—C45	1.453 (3)
C22—N21	1.355 (2)	N41—C46	1.457 (3)
C22—C23	1.411 (3)	C45—H45A	0.9800
C23—C24	1.368 (3)	C45—H45B	0.9800
C23—H23	0.9500	C45—H45C	0.9800
C24—H24	0.9500	C46—H46A	0.9800
N21—C26	1.452 (3)	C46—H46B	0.9800
N21—C25	1.457 (3)	C46—H46C	0.9800
			
N1—Mn1—N2	92.28 (7)	N21—C25—H25B	109.5
N1—Mn1—N3	89.70 (8)	H25A—C25—H25B	109.5
N2—Mn1—N3	177.98 (7)	N21—C25—H25C	109.5
N1—Mn1—N4	178.78 (7)	H25A—C25—H25C	109.5
N2—Mn1—N4	88.76 (8)	H25B—C25—H25C	109.5
N3—Mn1—N4	89.26 (8)	N21—C26—H26A	109.5
N1—Mn1—N20	92.86 (6)	N21—C26—H26B	109.5
N2—Mn1—N20	92.57 (7)	H26A—C26—H26B	109.5
N3—Mn1—N20	87.73 (7)	N21—C26—H26C	109.5
N4—Mn1—N20	87.73 (6)	H26A—C26—H26C	109.5
N1—Mn1—N10	91.48 (6)	H26B—C26—H26C	109.5
N2—Mn1—N10	91.46 (7)	C34—N30—C30	120.9 (2)
N3—Mn1—N10	88.08 (7)	C34—N30—H30	119.6
N4—Mn1—N10	87.86 (6)	C30—N30—H30	119.6
N20—Mn1—N10	173.95 (6)	N30—C30—C31	121.2 (2)
C1—N1—Mn1	168.73 (18)	N30—C30—H30A	119.4
N1—C1—S1	178.8 (2)	C31—C30—H30A	119.4
C2—N2—Mn1	163.48 (19)	C30—C31—C32	120.0 (2)
N2—C2—S2	179.9 (3)	C30—C31—H31	120.0
C3—N3—Mn1	150.20 (18)	C32—C31—H31	120.0
N3—C3—S3	179.7 (2)	N31—C32—C31	121.73 (18)
C4—N4—Mn1	147.24 (19)	N31—C32—C33	121.70 (18)
N4—C4—S4	179.3 (2)	C31—C32—C33	116.57 (19)
C14—N10—C10	115.37 (17)	C34—C33—C32	120.0 (2)
C14—N10—Mn1	123.78 (13)	C34—C33—H33	120.0
C10—N10—Mn1	120.85 (13)	C32—C33—H33	120.0
N10—C10—C11	124.47 (19)	N30—C34—C33	121.4 (2)
N10—C10—H10	117.8	N30—C34—H34	119.3
C11—C10—H10	117.8	C33—C34—H34	119.3
C10—C11—C12	120.1 (2)	C32—N31—C36	121.46 (18)
C10—C11—H11	120.0	C32—N31—C35	121.58 (17)
C12—C11—H11	120.0	C36—N31—C35	116.95 (19)
N11—C12—C13	123.0 (2)	N31—C35—H35A	109.5
N11—C12—C11	121.4 (2)	N31—C35—H35B	109.5
C13—C12—C11	115.61 (18)	H35A—C35—H35B	109.5
C14—C13—C12	119.70 (19)	N31—C35—H35C	109.5
C14—C13—H13	120.2	H35A—C35—H35C	109.5
C12—C13—H13	120.2	H35B—C35—H35C	109.5
N10—C14—C13	124.79 (19)	N31—C36—H36A	109.5
N10—C14—H14	117.6	N31—C36—H36B	109.5
C13—C14—H14	117.6	H36A—C36—H36B	109.5
C12—N11—C16	121.6 (3)	N31—C36—H36C	109.5
C12—N11—C15	120.2 (2)	H36A—C36—H36C	109.5
C16—N11—C15	118.2 (2)	H36B—C36—H36C	109.5
N11—C15—H15A	109.5	C40—N40—C44	120.7 (3)
N11—C15—H15B	109.5	C40—N40—H40A	119.7
H15A—C15—H15B	109.5	C44—N40—H40A	119.7
N11—C15—H15C	109.5	C41—C40—N40	121.3 (3)
H15A—C15—H15C	109.5	C41—C40—H40	119.4
H15B—C15—H15C	109.5	N40—C40—H40	119.4
N11—C16—H16A	109.5	C40—C41—C42	120.1 (2)
N11—C16—H16B	109.5	C40—C41—H41	120.0
H16A—C16—H16B	109.5	C42—C41—H41	120.0
N11—C16—H16C	109.5	N41—C42—C41	121.6 (2)
H16A—C16—H16C	109.5	N41—C42—C43	122.07 (19)
H16B—C16—H16C	109.5	C41—C42—C43	116.3 (2)
C24—N20—C20	115.08 (16)	C44—C43—C42	120.5 (2)
C24—N20—Mn1	124.47 (13)	C44—C43—H43	119.7
C20—N20—Mn1	120.43 (13)	C42—C43—H43	119.7
N20—C20—C21	124.96 (18)	C43—C44—N40	121.1 (3)
N20—C20—H20	117.5	C43—C44—H44	119.4
C21—C20—H20	117.5	N40—C44—H44	119.4
C20—C21—C22	119.79 (18)	C42—N41—C45	121.17 (18)
C20—C21—H21	120.1	C42—N41—C46	121.77 (19)
C22—C21—H21	120.1	C45—N41—C46	117.0 (2)
N21—C22—C23	123.08 (18)	N41—C45—H45A	109.5
N21—C22—C21	121.45 (18)	N41—C45—H45B	109.5
C23—C22—C21	115.47 (17)	H45A—C45—H45B	109.5
C24—C23—C22	119.75 (18)	N41—C45—H45C	109.5
C24—C23—H23	120.1	H45A—C45—H45C	109.5
C22—C23—H23	120.1	H45B—C45—H45C	109.5
N20—C24—C23	124.95 (18)	N41—C46—H46A	109.5
N20—C24—H24	117.5	N41—C46—H46B	109.5
C23—C24—H24	117.5	H46A—C46—H46B	109.5
C22—N21—C26	121.37 (19)	N41—C46—H46C	109.5
C22—N21—C25	120.15 (18)	H46A—C46—H46C	109.5
C26—N21—C25	118.46 (18)	H46B—C46—H46C	109.5
N21—C25—H25A	109.5		

### Hydrogen-bond geometry (Å, º)

**Table:**

*D*—H···*A*	*D*—H	H···*A*	*D*···*A*	*D*—H···*A*
N30—H30···S4	0.88	2.36	3.217 (2)	165
N40—H40*A*···S3^i^	0.88	2.35	3.213 (3)	166

Symmetry code: (i) *x*+1, *y*+1, *z*+1.
